# Retraction: GNA14 silencing suppresses the proliferation of endometrial carcinoma cells through inducing apoptosis and G2/M cell cycle arrest

**DOI:** 10.1042/BSR20180574_RET

**Published:** 2026-05-06

**Authors:** Bo Hang, Jing Wang, Xiao Lv, Feixue Xu, Min Wei, Cuiping Liu, Yongxiu Yang

**Affiliations:** Department of Obstetrics and Gynecology, The First Hospital of Lanzhou University, Lanzhou 730000, Gansu, P.R. China

**Keywords:** apoptosis, cell cycle arrest, endometrial carcinoma, GNA14, proliferation

This article is being retracted from *Bioscience Reports* at the request of the Editor-in-Chief and the Editorial Board. This follows the receipt of a notification from a reader, alerting the Editorial Board to similarities between this article and one published by a different author group.

The Editorial Office identified two further instances of similarity with other articles. Specifically, these include the following:

- The Figure 2C GNA14 bands from this article appear highly similar to the Figure 6B C-RAF bands in Gong et al. (2015) DOI: 10.18632/oncotarget.4624
- The Figure 6D Caspase-3 bands from this article appear highly similar to the Figure 2B MRPL42 bands (after a horizontal flip) from Hao et al. (2018) DOI: 10.1042/BSR20171456
- The Figure 6D GAPDH bands appear highly similar to the Figure 2D GAPDH bands from Bai et al. (2018) DOI: 10.1042/BSR20180454.

The authors were contacted with regard to the concerns but were unable to provide raw data. Given the extent of the issues raised, the Editorial Board stands by the decision to retract the article. The authors disagree to the retraction.

